# 

**Published:** 1914-08-01

**Authors:** 


### Medical Appointments.

Mr. G. H. Monrad-Krohn has been appointed sixth assistant medical officer at Bexley Mental Hospital.

Mr. D. A. Macpherson, fifth assistant medical officer at Long Grove Mental Hospital, having resigned his appointment, Mr. H. W. Hills, the sixth assistant medical officer, has been promoted to succeed him.

The following have been appointed clinical assistants to the out-patients at the West End Hospital for Diseases of the Nervous System :—Physicians : Henry Braund, M.R.C.S., L.R.C.P. ; H. B. Newham, M.R.C.S., L.R.C.P., D.P.H. (Camb.), director, London School of Tropical Medicine, Albert Dock; and J. P. Park Inglis, M.B., Ch.B., assistant medical officer, Fountain Temporary Asylum. The following have been reappointed clinical assistants : Jas. Hutchison, M.D. ; J. Russell Higson, M.B., C.M. ; Julian Landman, M.D. ; J. W. Pugh, M.D.

### Interesting Function at the West End Hospital.

A ceremony of some interest took place on Thursday afternoon, July 23, at the West End Hospital for Diseases of the Nervous System, when the Savill Medal and Prize were presented for the first time, the winner being Dr. T. Knowles Boney. In the absence of Mr. Leonard Brassey, M.P., the chair was taken by Dr. Harry Campbell, vice-chairman and senior physician of the hospital. Dr. Campbell introduced Mrs. Agnes Savill, M.D., and Mrs. Hardy, the former of whom presented the medal and cheque, and the latter copies of the late Dr. Savill's three books. The medal and prize were founded in memory of the late Dr. T. D. Savill, a former physician of the hospital, who lost his life accidentally in Algiers in 1910. The medal bears the portrait of Dr. Savill on its face, while on the reverse " Medicine " is symbolised by a figure of Hygieia, daughter or wife of Aesculapius, feeding a serpent from a cup. Hygieia is said to have originally been the Goddess of Physical Health, but latterly to have been regarded as giver or protectress of mental health, and so specially fitted to commemorate a researcher like Dr. Savill. The motto

" Life is but a little holding
Lent to do a mighty labour,"

taken from George Meredith, was a favourite of Dr. Savill's, and may be said to embody his concept of what a physician's ideal should be. The Savill Lecture is to be given in November by Dr. Harry Campbell, on a day to be announced later.

Rates of Subscription: Twelve months, 6/6; Six months, 4/-; Three months, 2/-, post free to any address in the United Kingdom. Abroad, Twelve months, 8/8; Six months, 5/-; Three months, 2/6, post free.—Address The Subscription Dept., "The Hospital," The Hospital Building, 28/29 Southampton Street, Strand, London W.C.

